# β-Amyloid species production and tau phosphorylation in iPSC-neurons with reference to neuropathologically characterized matched donor brains

**DOI:** 10.1093/jnen/nlae053

**Published:** 2024-06-14

**Authors:** Derek H Oakley, Mirra Chung, Sara Abrha, Bradley T Hyman, Matthew P Frosch

**Affiliations:** Harvard Medical School, Boston, MA, United States; C.S. Kubik Laboratory for Neuropathology, Boston, MA, United States; Massachusetts Alzheimer’s Disease Research Center, Charlestown, MA, United States; Department of Pathology, Massachusetts General Hospital, Boston, MA, United States; Harvard Medical School, Boston, MA, United States; Department of Neurology, Massachusetts General Hospital, Boston, MA, United States; Harvard Medical School, Boston, MA, United States; Department of Neurology, Massachusetts General Hospital, Boston, MA, United States; Harvard Medical School, Boston, MA, United States; Massachusetts Alzheimer’s Disease Research Center, Charlestown, MA, United States; Department of Neurology, Massachusetts General Hospital, Boston, MA, United States; Harvard Medical School, Boston, MA, United States; C.S. Kubik Laboratory for Neuropathology, Boston, MA, United States; Massachusetts Alzheimer’s Disease Research Center, Charlestown, MA, United States; Department of Pathology, Massachusetts General Hospital, Boston, MA, United States

**Keywords:** Alzheimer disease, Beta amyloid, iPSCs, Neurodegeneration, Tau

## Abstract

A basic assumption underlying induced pluripotent stem cell (iPSC) models of neurodegeneration is that disease-relevant pathologies present in brain tissue are also represented in donor-matched cells differentiated from iPSCs. However, few studies have tested this hypothesis in matched iPSCs and neuropathologically characterized donated brain tissues. To address this, we assessed iPSC-neuron production of β-amyloid (Aβ) Aβ40, Aβ42, and Aβ43 in 24 iPSC lines matched to donor brains with primary neuropathologic diagnoses of sporadic AD (sAD), familial AD (fAD), control, and other neurodegenerative disorders. Our results demonstrate a positive correlation between Aβ43 production by fAD iPSC-neurons and Aβ43 accumulation in matched brain tissues but do not reveal a substantial correlation in soluble Aβ species between control or sAD iPSC-neurons and matched brains. However, we found that the ApoE4 genotype is associated with increased Aβ production by AD iPSC-neurons. Pathologic tau phosphorylation was found to be increased in AD and fAD iPSC-neurons compared to controls and positively correlated with the relative abundance of longer-length Aβ species produced by these cells. Taken together, our results demonstrate that sAD-predisposing genetic factors influence iPSC-neuron phenotypes and that these cells are capturing disease-relevant and patient-specific components of the amyloid cascade.

## Introduction

Induced pluripotent stem cell-derived neurons (iPSC-neurons) are the basis of numerous in vitro models of Alzheimer disease (AD) and other neurodegenerative disorders.^1–3^ A basic assumption underlying these models is that disease-relevant pathologies present in brain tissue are also present in iPSC-neurons. However, few studies have had access to matched sets of iPSCs and neuropathologically characterized donated brain tissues to validate this assumption. At the Massachusetts Alzheimer’s Disease Research Center (MADRC), we have developed a cohort of iPSC lines and matched donated brains to address this issue. This work represents a step toward constructing patient-specific models of AD that explore the relationship between iPSC-neurons and matched donor brains.

Extracellular β-amyloid (Aβ) aggregation and intraneuronal neurofibrillary tangle formation are the hallmark pathologies of AD.^4^^,^^5^ Alterations in Aβ processing, specifically the generation of longer-length more amyloidogenic Aβ species such as Aβ42 and Aβ43, are believed to predispose to Aβ plaque formation and drive pathologic tau phosphorylation and subsequent aggregation.^6^ Thus, understanding factors that influence Aβ processing in the human brain may shed light on individual risk for AD. Familial AD (fAD)-causing mutations in genes such as *PSEN1* and *APP* are well studied in this context and are known to drive mutation-specific changes in the stoichiometry of Aβ processing, both in cell-based in vitro models and in vivo.^7–9^ It is less clear how this relates to sporadic AD (sAD) human neuronal Aβ production or Aβ deposition in human brain tissues. Furthermore, the relationship between endogenous variation in Aβ processing and subsequent pathologic tau phosphorylation has been studied in very few iPSC-neuron cohorts with neuropathologic confirmation of diagnosis.^1^^,^^10^ Investigating this relationship in human neurons can be used to evaluate the amyloid hypothesis and may be a step toward understanding patient-specific variation in AD predisposition.

This work first assesses the relationship between Aβ accumulation in donated brain tissues and Aβ production in matched iPSC-neurons using a small group of fAD donors. Then, these analyses are expanded to a larger cohort consisting of neuropathologically confirmed control, fAD, sAD, Down syndrome (DS), and frontotemporal lobar degeneration with Tau pathology cases (FTLD-tau). We also evaluate the association of primary neuropathologic diagnosis and ApoE genotype with Aβ species production. Subsequently, pTau/total tau ratio in iPSC-neurons is determined by Western blot for this group and evaluated in the context of primary neuropathologic diagnosis. Because the amyloid hypothesis places elevation in pTau downstream of alterations in Aβ processing, we examine the relationship between iPSC-neuron pTau/total tau ratio and iPSC-neuron secreted Aβ42/40 ratio in control, sAD, fAD, and DS donors. Ultimately, this work will help to elucidate phenotypes of AD present in iPSC-neurons and their relationship to donor-matched brain tissues.

## Methods

### Tissue donation and neuropathologic characterization

All human brain tissues were obtained from the MADRC (P30AG062421). Autopsy evaluation of each donated brain was carried out by a board-certified neuropathologist at the MADRC and was consistent with current guidelines for evaluation of neurodegenerative disease,^4^^,^^5^ including immunohistochemistry for Aβ, tau, α-synuclein, and phosphorylated TDP-43, as well as assessment for vascular disease.

### Sequence validation of familial disease

iPSC cultures were grown to sub-confluence and DNA was extracted using Qiagene DNeasy Blood & Tissue Kit (cat. 69504). Sequence validation for *PSEN1*, *APP*, and *MAPT* genes was performed by commercial Sanger sequencing (Genewiz).

### Fibroblast culture and iPSC generation

Fibroblasts were cultured as previously described via explant culture on Corning CellBind flasks and growth in culture medium consisting of DMEM, 10% fetal bovine serum, l-glutamine (2 mM), and antibiotic-antimycotic (1×) (Gibco, ThermoFisher, Waltham, MA, USA).^10^

All iPSCs were commercially generated from primary fibroblast cultures using mRNA reprogramming (Cellular Reprogramming, Inc., Pasadena, CA).^11^^,^^12^ iPSC cultures were maintained in mTeSR Plus media (StemCell Technologies, Vancouver, CA) on Matrigel-coated dishes (Corning, Corning, NY, USA). Routine passaging was performed using Accutase (Gibco, ThermoFisher). Cell line identity was confirmed via STR profiling with comparison to matched reference brain tissue using the CLA IdentiFiler Plus system (ThermoFisher).

### iPSC-neuron differentiation and culture

Viral neurogenin-2 (NGN2) differentiations were performed as described previously with dual SMAD inhibition.^10^^,^^13–17^ For iPSC-lines with NGN2 PiggyBAC integration, a doxycycline inducible NGN2-2A-PuroR PiggyBAC vector (generously provided by the Li-Huei Tsai laboratory at the Massachusetts Institute of Technology) was stably integrated into polyclonal iPSC cultures as follows: One day prior to transfection, iPSCs were passaged as single cells using enzymatic dissociation with Accutase in the presence of 1 µM Thiazovivin and plated at 1-2 M cells per well on 6-well plates. One day after plating, iPSCs were lipofected with the NGN2 PiggyBAC plasmid (2 µg) as well as a transposase expression vector (pEF1a-hyPBase, 1.5 µg) using Lipofectamine Stem (ThermoFisher STEM0003) according to the manufacturer’s instructions and in the presence of 1 µM Thiazovivin. One day after transfection, media was changed and selection was begun with blasticidin (5 µg/mL). Three days after the transfection, the media was changed again. Thiazovivin was maintained until 5 days post-transfection.

Subsequently, iPSCs were differentiated into neurons using doxycycline induction of the integrated human NGN2 gene. Differentiation was performed as follows: at day −1, iPSCs were passaged as single cells following accutase digestion and plated 1.0-1.25 × 10^6^ cells per well of a 1× Matrigel coated 6-well plate in mTeSR1 plus supplemented with 1 µM Thiazovivin and 2.0 µg/mL doxycycline. Doxycycline was maintained for the duration of differentiation. On day 0, media was switched to DMEM/F-12 supplemented with Glutamax, Non-Essential Amino Acids, and N2 supplement (Differentiation Media -B27). On day 2, the media was changed to Differentiation Media supplemented with B27 and 10 ng/mL of BDNF and NT3 (Complete Differentiation Media). On day 4, differentiating cells were passaged using Accutase digestion and plated in Matrigel-coated 96 well plates, 6-well plates, or frozen in mFreSR (StemCell Technologies) freezing media for future differentiation. At day 5, media was changed to Neurobasal supplemented with doxycycline, B27, 2% horse serum, 5 µg/mL Puromycin, and 10 ng/mL each of BDNF, NT3, and GDNF. Subsequently, 50% of media changes were performed every other day until day 14. After day 14, 50% of media changes were performed every 2-3 days. For experiments involving secreted AB measurement, media was allowed 7 days of incubation with cells prior to harvest.

### Enzyme-linked immunosorbent assay

Enzyme-linked immunosorbent assay (ELISA) measurements of Aβ species were run according to the manufacturer’s instructions as previously described.^10^ Cell media was aspirated, centrifuged to remove debris, and frozen at –80°C prior to measurements. Supernatant was diluted 1:5 prior to measurement of Aβ40 and Aβ42, and run undiluted for Aβ43. Detection reagents were: Human Aβ 1-40 (298-64601, FUJIFILM Wako Chemicals U.S.A. Corporation, Richmond, VA), human Aβ 1-42 (WAKO 296-64401), and human Aβ 1-43 (27710, Immuno-Biological Laboratories, Inc., Minneapolis, MN). Assay plates were read on a Wallac Victor2 at 450 nm (Perkin Elmer, Waltham, MA, USA). Correction for batch effects in ELISA data was performed via a median scaling in R.

### Immunocytochemistry

iPSCs and iPSC-neurons were fixed with 4% paraformaldehyde in PBS for 30 min at room temperature (RT). Cells were washed with PBS and permeabilized for 15 min at RT with 0.1% Triton X-100. Cells were then washed again with PBS and blocked for 1 h at RT with PBS^−/−^, 5% NGS (Figure S2) or Intercept Blocking Buffer-PBS (Li-COR 927-70001, Figures S1 and S3). Primary antibodies were applied O/N at 4°C in a blocking buffer. Secondary antibodies were applied for 1 hour at RT at 1:500 in the blocking buffer. DAPI (1 µg/mL, Roche) was applied for 15 min at RT in PBS^−/−^ or blocking buffer. Stained cells were imaged on a Biotek Cytation 5 microscope (Biotek Instruments Inc., Winooski, VT). Antibodies used: Oct4 (1:1000, Abcam ab19857) Sox2 (1:1000, Abcam ab97959), Nanog (1:250, Abcam ab21624), PHF1 (Davies Laboratory 1:200), Tuj1/BIII Tubulin (1:500, Aves TUJ1), and Map2 (1:250, Abcam ab32454).

### Western blot

As described previously, iPSC-neurons were harvested at D28 of differentiation on ice into ice-cold RIPA buffer with protease inhibitor (Cell Signaling, Danvers, MA, USA, Cat #5871S) using a cell scraper.^10^ After scraping, cells were lysed with 10 passes using insulin syringes and pelleted for 10 min at 10 kg and 4°C. The resulting supernatant and pellets were saved at –80°C. PBS-soluble brain extracts from the frontal cortex (Brodmann area 9 [BA9]) were prepared as described previously by dounce-homogenization in ice-cold PBS^−/−^ with protease inhibitor (Cell Signaling, Cat #5871S) followed by centrifugation at 10,000 g for 10 min.^10^

Western blots were performed on 20 µg samples of soluble supernatants using the Invitrogen NuPage Novex Gel System according to the manufacturer’s instructions (ThermoFisher). Fluorescent secondary antibodies were used at a concentration of 1:5000, according to the manufacturer’s instructions. Blots were imaged on a LI-COR Odyssey system and analyzed using LI-COR Image Studio (LI-COR Biosciences, Lincoln, NE). Antibodies used: AT8 (Phospho-Tau, Ser202, Thr205, 1:1000, Invitrogen/ThermoFisher MN1020), and D5 (Total Tau-D5D8N, 1:1000, Cell Signaling 43894S).

### Statistical methods

Calculation of standard deviation, 2-tailed Student *t*-tests, and Pearson linear correlation coefficients were performed using R.

## Results

### Generation of iPSCs from a panel of brain donors

Derivation of primary fibroblast lines at autopsy was performed via explant culture as described previously.^10^ A group of primary fibroblast lines was subsequently reprogrammed to iPSCs using a non-integrating mRNA approach (see Methods). This group of 24 iPSC lines is composed of controls, and patients with sAD, fAD, DS, and several forms of FTLD-tau (Table 1). Each of the 24 iPSC lines appropriately expresses the pluripotency markers Oct4, Sox2, and Nanog as assessed by immunohistochemistry (Figure S1).

**Table 1.** Donor characteristics.

Donor	NPDX1	NPDX2	Age	Sex	ApoE	BraakNFT	Thal	CERAD	Gene
2068	Control	CVD	79	Female	3/3	II	0	0	NA
2191	Control	CVD	87	Male	2/3	II	3	1	NA
2380	Control	CVD	89	Female	3/3	I	0	0	NA
2057	AD		85	Male	3/3	V	4	2	NA
2021	AD	CVD	85	Female	3/4	V	4	2	NA
2058	AD	CVD	90+	Male	3/4	V	4	2	NA
2178	AD	CVD	54	Female	3/3	VI	4	3	NA
2232	AD	CVD	65	Female	3/3	V	5	3	NA
2210	AD	CVD	66	Female	3/3	VI	5	3	NA
2037	AD	CVD	73	Female	3/4	VI	5	3	NA
2215	AD	CVD/CAA	70	Male	3/3	VI	5	2	NA
2027	AD	LBD/DLB	76	Male	3/4	V	4	3	NA
2243	fAD	CAA	58	Male	2/3	VI	5	3	PS1 P264L
2048	fAD	CAA	53	Female	3/3	VI	3	3	PS1 L435F
2165	fAD	CVD/CAA	59	Female	3/3	VI	5	3	PS1 H163R
2265	fAD	LBD/DLB	52	Male	3/4	VI	5	3	APP V717I
2379	DS	CAA	60	Female	2/4	VI	5	3	Trisomy 21
2218	DS	CAA	70	Female	3/4	VI	5	3	Trisomy 21
2409	DS	CVD	54	Male	2/3	I	3	1	Trisomy 21
2410	DS	CVD	68	Male	3/3	VI	5	3	Trisomy 21
2109	FTLD	ADNC	71	Female	3/3	IV	2	0	MAPT P301L
2041	PiD		73	Female	3/3	0	0	0	NA
2043	PSP	CVD	73	Male	3/3	0	0	0	NA
2326	CBD	CVD	61	Male	3/3	0	0	0	MAPT P301L

Abbreviations: AD = sporadic AD (sAD, AD), ADNC = Alzheimer disease neuropathologic changes, ApoE = ApoE genotype, BraakNFT = Braak and Braak neurofibrillary tangle stage, CAA = cerebral amyloid angiopathy, CBD = corticobasal degeneration, CERAD = neuritic plaque density score, CVD = cerebrovascular disease, DS = Down syndrome, fAD = familial AD, FTLD = frontotemporal lobar degeneration with Tau pathology (FTLD-Tau), LBD/DLB = Lewy body disease, NPDX1 = primary neuropathologic diagnosis, PiD = Pick disease, PSP = progressive supranuclear palsy, NPDX2 = secondary neuropathologic diagnosis, Thal = Thal beta amyloid stage.

### Aβ43 immunohistochemistry in donated fAD brains predicts matched iPSC-neuron Aβ43 secretion

**Figure 1.**
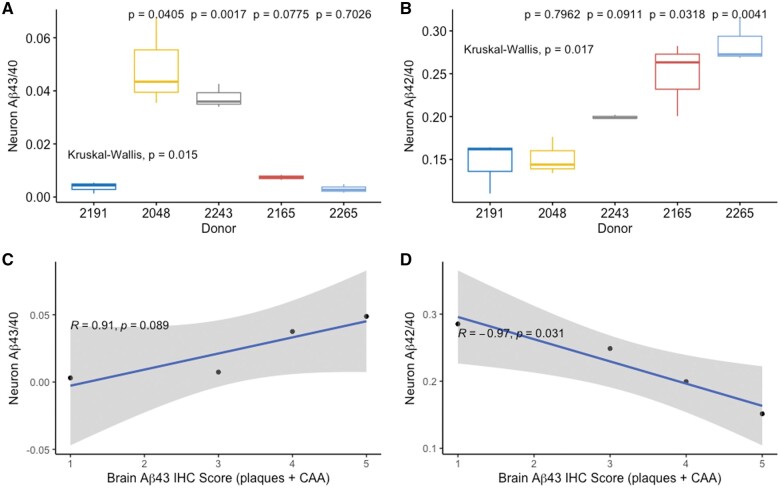
Brain Aβ IHC score predicts Aβ43/40 ratio in fAD iPSC-neurons. (A) Aβ43/40 ratio for neurons differentiated from each iPSC line. *P*-values for comparison against the control line 2191 are shown above. (B) Aβ42/40 ratio for neurons differentiated from each iPSC line. *N* = 3 neuronal differentiations for each line. (C, D) Comparison of neuronal Aβ43/40 ratio (C) and Aβ42/40 ratio (D) for each line to matched brain Aβ43 IHC score derived from reference 10. Brain Aβ43 IHC scores are as follows: 2191 [NA], 2265 [1], 2165 [3], 2243 [4], and 2048 [5].

Our prior work indicates that Aβ43 species are elevated in fAD patient brain tissues and in iPSC-neurons carrying the PSEN1 L435F mutation.^10^ To extend these results, we sought to assess the correlation between the level of Aβ43 present in fAD patient frontal cortex (BA9) and that produced by matched iPSC-neurons in 4 patients with fAD. Mutations present in the group of 4 fAD lines are PSEN1 L435F, PSEN1 H163R, PSEN1 P264L, and APP V717I. In our prior study, we scored the level of Aβ43 present in neocortical plaques and in cerebral amyloid angiopathy (CAA) on a scale of 1-3 for each measure. These 2 measures were added to form an “Aβ43 score” for the 4 fAD cases in the prior study (Figure 1).

Subsequently, Aβ species were measured in matched iPSC-neurons. Using a lentiviral approach in conjunction with antibiotic selection, 1 control and 4 fAD lines were made to stably express a doxycycline-inducible NGN2 gene allowing rapid differentiation into layer II/III cortical neurons over a period of approximately 14 days.^10^^,^^16–18^ At 28 days of differentiation, media was collected and cells were fixed. Cultures were then stained for neuronal markers to verify Neuronal differentiation (Figure S2). Aβ40, Aβ42, and Aβ43 species were measured in the culture media by ELISA. Aβ42/40 and Aβ43/40 ratios were then plotted against the semiquantitative Aβ43 score determined in our prior work^10^ (Figure 1). Our results show a positive relationship between the Aβ43 score and iPSC-neuron secreted Aβ43/40 ratio (*R* = 0.91, *P* = .089, Figure 1) and a negative relationship between the Aβ43 score and iPSC-neuron secreted Aβ42/40 ratio (*R* = −0.97, *P* = .031, Figure 1). The negative relationship between the Aβ43 score and iPSC-neuron secreted Aβ42/40 ratio may reflect the production of Aβ43 at the expense of Aβ42 in these cultures. In addition, each of the fAD lines showed a significantly higher Aβ43/40 or Aβ42/40 ratio compared to the control line (Figure 1). These data indicate that in the context of a strong genetic driver such as a mutation in PSEN1 or APP, iPSC-neuron Aβ production is altered and, to some extent, reflects Aβ43 deposition in matched brains.

### Soluble Aβ species measurement in a panel of donated brains and matched iPSC-neurons

Given that the vast majority of patients with AD do not have dominantly inherited disease, we then sought to determine the relationship of iPSC-neuron Aβ secretion to Aβ deposition in the brains of patients with sAD. To accomplish this, 9 sAD iPSC lines were generated (Table 1). We also chose to generate additional control and DS lines as well as a group of FTLD-tau lines to serve as an outgroup with Aβ-independent neurodegenerative tauopathy (Table 1). The lentiviral-NGN2 approach used to generate iPSC-neurons above, while efficient, requires concurrent chemical dual SMAD inhibition and is somewhat vulnerable to silencing resulting in a reduction in the percent neuronal yield over time. In order to work with the larger panel of 24 iPSC lines, a more efficient protocol using stable PiggyBAC transposon-mediated integration of the inducible NGN2 cassette was implemented.^13^^,^^19–21^ Each iPSC line was stably integrated with a doxycycline-inducible NGN2 construct using transposase and differentiated to day 28 post-mitotic neurons (Figure S3, see Methods). Media was harvested as above to 5 biological replicates. Due to the change in neuronal differentiation procedures, new PiggyBAC inducible NGN2 stable isolates of the initial fAD and control lines above were re-differentiated in this set of experiments (Figure S3). Neuronal clustering and detachment complicated our efforts to perform quantitative histologic metrics on these cultures. Future work using improved cell attachment substrates or possibly astrocyte feeder layers may be helpful in this respect.

**Figure 2.**
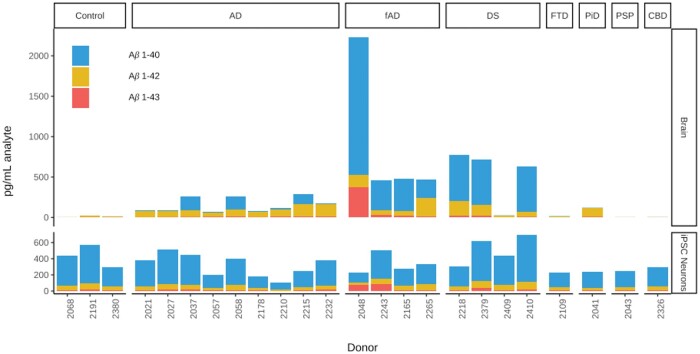
Aβ species production from iPSC-neurons and matched donor frontal cortex. Mean Aβ40, Aβ42, and Aβ43 levels, as determined by ELISA are depicted in pg/mL of brain lysate (BA9) and neuronal culture media. Column facets represent primary neuropathologic diagnosis (NPDX1) and rows indicate source of material (brain or iPSC-neuron culture media/supernatant).

Aβ40, Aβ42, and Aβ43 species were then measured in the culture media and donor-matched frontal cortex homogenates by ELISA (BA9, Figure 2). As expected, sAD, fAD, and DS cases showed increased levels of Aβ present in the frontal cortex compared to control and FTLD-Tau cases (Figure 2). iPS-neuron Aβ production was less clearly related to diagnosis (Figure 2). Interestingly, one case in the DS cohort displayed low levels of soluble Aβ species in the frontal cortex, consistent with the mild AD neuropathologic changes present at autopsy and in the context of an ApoE 2/3 genotype (Table 1, Figure 2). On average, iPSC-neuron soluble Aβ levels were not significantly different in the disease categories (fAD, sAD, DS, FTLD) versus the control lines (Figure 4). However, there was a nonsignificant trend toward higher overall Aβ species production in DS lines (Figure 4).

**Figure 3.**
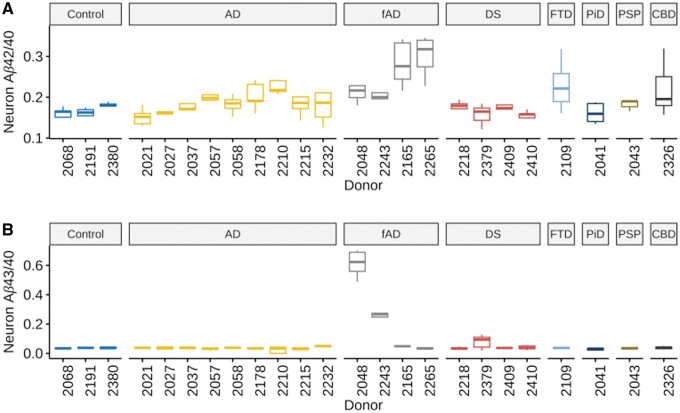
Aβ42/40 and Aβ43/40 species ratios in cultures of iPSC-neurons. (A) Aβ42/40 ratio in neuronal culture media. (B) Aβ43/40 ratio in neuronal culture media. Column facets represent primary neuropathologic diagnosis (NPDX1, *n* = 5 neuronal differentiations). fAD measurements are independent from those in Figure 1 and maintain a consistent relationship to Brain Aβ IHC score. Outliers are not plotted for clarity.

**Figure 4.**
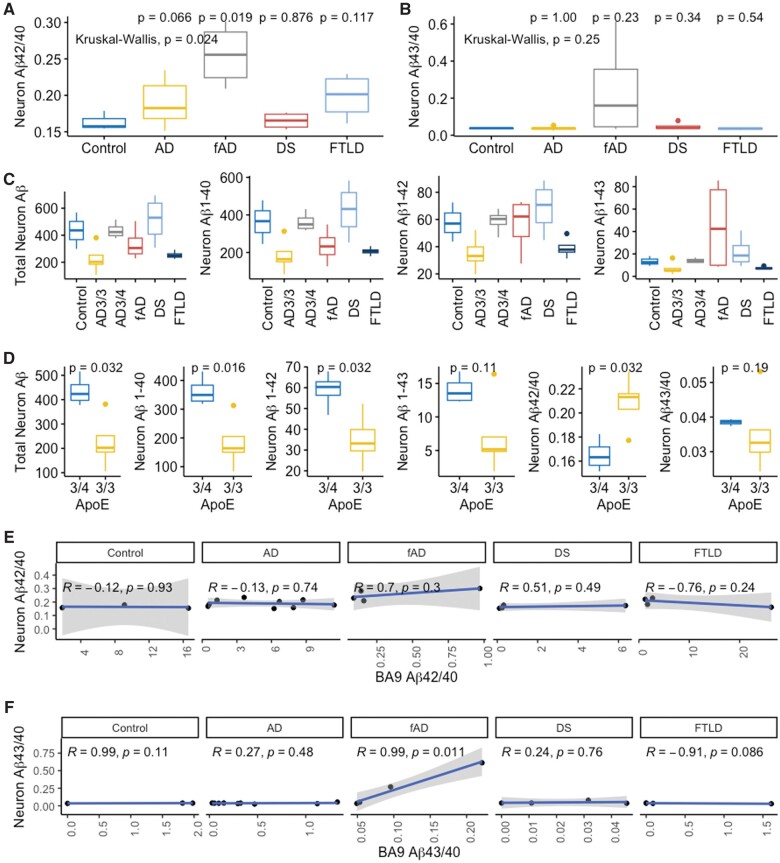
Aβ species production by primary neuropathologic diagnosis, ApoE genotype, and compared to donor-matched frontal cortex. Aβ42/40 (A) and Aβ43/40 (B) ratio for Control, AD, fAD, DS, and FTLD groups. FTLD comprises PiD, PSP, CBD, and FTLD-tau cases. *P*-values are listed above in the plot for comparison to the control group. (C) Total Aβ species levels in culture supernatant for each diagnostic category, measured by ELISA. Total Aβ = Aβ40 + 42 + 43. AD3/3 = AD with ApoE 3/3 genotype, AD3/4 = AD with ApoE 3/4 genotype. No significant differences vs control were present (*P* > .05 for each comparison). (D) Aβ species levels and ratios for ApoE 3/3 and 3/4 sAD cases. (E) Aβ42/40 ratio in iPSC-neurons compared to the donor-matched frontal cortex (BA9). (F) Aβ43/40 ratio in iPSC-neurons compared to donor-matched frontal cortex.

Aβ42/40 and Aβ43/40 ratios were then calculated for each iPS line (Figure 3). The relative relationships of Aβ42/40 and Aβ43/40 ratio for the four fAD iPSC lines differentiated via the NGN2 PiggyBAC approach were similar to that derived from the lentiviral-based differentiation protocol presented above, supporting the notion that these 2 techniques are roughly comparable (Figures 1 and 3). Overall, Aβ42/40 ratio was variable across lines (Figure 3) and was significantly higher in the fAD disease category compared to controls (*P* = .019, Figure 4). Similar results were observed for Aβ43/40 ratio with this metric being very low in non-fAD iPSC lines (Figure 3). When analyzed as a group, the 4 FTLD lines (Pick disease [PiD], progressive supranuclear palsy [PSP], corticobasal degeneration [CBD], and FTLD-Tau) did not show significantly different Aβ42/40 or Aβ43/40 ratios compared to controls (*P* = .117 and .54, respectively). This result suggests that the control group is not likely to be severely biased toward donors with unusually low ratios in these metrics (Figure 4). A nonsignificant trend toward lower overall Aβ production was present in the FTLD lines (Figure 4). This may be due to the ApoE3/3 genotype present in each of these lines as the raw Aβ production levels are similar to those seen in ApoE3/3 AD neurons (see below).

Aβ production in iPSC-neurons was then directly compared to soluble Aβ levels in the matched donor brains. Aβ42/40 ratios were not correlated in iPSC-neurons and matched patient brain tissues in the AD, fAD, DS, or Control categories (Figure 4). Aβ43/40 ratios were positively correlated between iPSC-neurons and brain tissues in the fAD group (*R* = 0.99, *P* = .011), but not in the control, sAD, or DS categories (Figure 4).

### Genetic risk factors are associated with altered Aβ species in AD iPSC-neurons

In addition, we evaluated the association of ApoE genotype with Aβ species secretion in the sAD group, which is comprised of 5 ApoE 3/3 and 4 ApoE 3/4 individuals (male to female ratio: ApoE3/3 = 0.66, ApoE3/4 = 1). In the sAD group ApoE 3/4 genotype was associated with higher production of Aβ40 (*P* = .016), Aβ42 (*P* = .032), and Total Aβ (Aβ40 + Aβ42 + Aβ43, *P* = .032), but not Aβ43 (*P* = .11) as well as a lower Aβ42/40 ratio (*P* = .032) (Figure 4). There was no significant effect of ApoE3/4 on Aβ43/40 ratio (*P* = .19) (Figure 4). sAD donor sex did not significantly influence Aβ species levels or ratios in iPSC-neurons (Figure S4).

### Aβ42/40 ratio correlates with pathologic tau phosphorylation in iPSC-neurons

**Figure 5.**
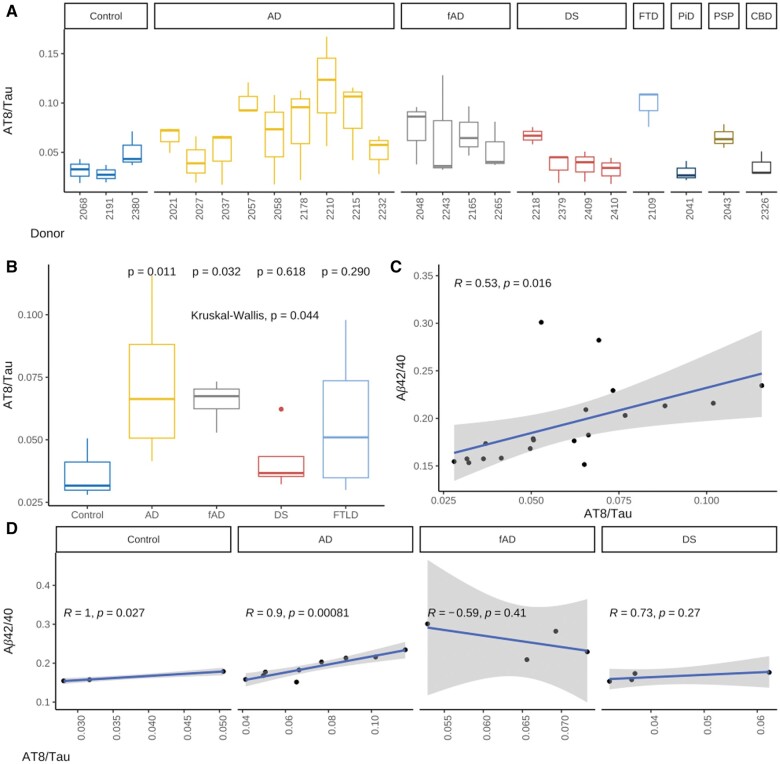
Relationship of pathologic tau phosphorylation to Aβ species production in iPSC-neurons. (A) Phospho-tau to total tau ratio (AT8/Tau) in neurons differentiated from each iPSC line measured by Western blot (*n* = 3 neuronal differentiations per line). (B) AT8/Tau ratio for each category of primary neuropathologic diagnosis. *P*-values are listed above in the plot for comparison to the control group. (C) Mean Aβ42/40 and AT8/tau ratios compared for control, AD, fAD, and DS lines. (D) Mean Aβ42/40 and AT8/tau data from C subset by primary neuropathologic diagnosis.

Finally, we sought to determine whether donor-dependent variation in Aβ processing might drive variation in tau phosphorylation, consistent with the amyloid hypothesis of AD pathogenesis. The 24-line iPSC cohort was differentiated into iPSC-neurons as above for 3 biological replicates. Cells were then harvested and a Western blot was performed for phosphorylated tau (pTau, AT8) and total tau protein. The AT8/total tau ratio was subsequently determined (Figure 5). As groups, both fAD iPSC-neurons and sAD iPSC-neurons showed a significantly higher pTau/Total tau ratio compared to controls (*P* = .032 and .011, respectively, Figure 5). pTau/total tau ratio was also positively correlated with Aβ42/40 ratio among the combined control, fAD, sAD, and DS lines (*R* = 0.53, *P* = .016, Figure 5). This positive correlation remained in the sAD (*R* = 0.99, *P* = .00081) and control (*R *= 1, *P* = .027) groups when the cell lines were subset by primary neuropathologic diagnosis (Figure 5). The low N in the control category (3 cell lines) is important to note in this context.

The FTLD-tau cases in our cohort are a neuropathologically heterogeneous group composed of FLTD-Tau MAPT P301L, CBD MAPT P301L, PSP, and PiD. Following in-aggregate analysis of tau and Aβ levels in this group, each individual line was compared to average control Aβ and pTau values (*n* = 3 differentiations for pTau/total tau and *n* = 5 for Aβ species). In this analysis, 2 of the four FTLD lines demonstrated significantly higher pTau/total tau ratios compared to the average of all 3 control lines (FTLD-Tau MAPT P301L and PSP, *P* = .016 and .025, respectively, Figure S5). One of the 4 FTLD lines (PSP) demonstrated a small, but significant increase in Aβ42/40 ratio compared to controls (Figure S5).

## Discussion

Understanding how iPSC-neurons resemble donor-matched adult brains is critical to using these cell models for disease-focused research. With this in mind, our group has generated a bank of primary dermal fibroblast lines from neurodegenerative disease patients and controls at the time of autopsy and brain donation. Subsequently, a full neurodegenerative disease-focused evaluation is performed on each donated brain resulting in a gold-standard diagnosis for each patient and the ability to control for concurrent neurodegenerative disease in subsequent studies. In this work, we focus on a subset of 24 iPSC lines, 23 of which have not been previously reported.^10^

We first sought to determine whether iPSC-neuron Aβ production would be reflective of brain Aβ levels for a group of fAD patients. Our prior work indicates that Aβ43 species are elevated in fAD patient brain tissues and in iPSC-neurons carrying the PSEN1 L435F mutation, so we focused first on this analyte.^10^ We find that fAD iPSC-neurons do indeed produce Aβ43/40 levels that reflect those in donated brains, measured by both immunohistochemical and biochemical methods. This finding is consistent with prior reports demonstrating that dominant AD-causing mutations result in characteristic Aβ processing alterations.^7^^,^^22^ We also found an inverse relationship between Aβ43 and Aβ42 levels, possibly reflecting the tendency of each line to produce Aβ43, an intermediate species in the production of Aβ40, at the expense of Aβ42.^8^^,^^23^

Conversely, we did not find a significant association between sAD iPSC-neuron Aβ secretion and soluble Aβ levels in matched patient brain tissues. These results suggest that in the absence of a strong genetic driver other factors influencing brain Aβ deposition and clearance may overwhelm the portion of Aβ variation that can be modeled using iPSC-neurons. Alternatively, insoluble Aβ species, which were not directly measured in our analysis, may make up this discrepancy.

Within the fAD group, we observed that some lines produced relatively more Aβ42 and others produced relatively more Aβ43. This finding is consistent with prior studies demonstrating that the PSEN1 H163R, PSEN1 P264L, and APP V717I mutations result in elevated Aβ42 species,^24–26^ whereas the PSEN1 L435F mutation causes preferential elevation in Aβ43 species.^10^^,^^27^ The relative Aβ processing effects of these specific fAD mutations have not been previously compared in a single study.

Within the sAD group, increased Aβ secretion was associated with the ApoE4 allele. Similar findings have been reported in iPSC-neurons exposed to exogenous sources of ApoE^28^^,^^29^ and in isogenic iPSC neurons with CRISPR-altered ApoE genotypes,^30^^,^^31^ but not to our knowledge in genetically diverse patient-derived iPSC lines. We also found decreased Aβ42/40 ratio in the ApoE4 group, in contrast to prior work.^30^ In human cerebrospinal fluid, the ApoE4 allele is associated with lower soluble Aβ42 levels, representing aggregation of Aβ42 species in the context of AD pathology.^32^ While it is possible that the lower Aβ42/40 ratio present in iPSC-neuronal cultures bearing the ApoE4 allele is secondary to the accumulation of Aβ42 in insoluble aggregates, this finding may also be a result of other drivers of AD pathology in the ApoE 3/3 AD iPSC cohort. The ApoE 3/3 AD donor group was pre-selected for neuropathologic AD in the absence of the most common genetic driver. Future work will evaluate the levels of insoluble and oligomeric Aβ species in this context. Altering ApoE genotype in our existing iPSC-lines is another important future confirmatory set of experiments.

Variation in Aβ processing has long been hypothesized to be a driver of AD pathology. Increased overall Aβ as well as increased Aβ42/40 and Aβ43/40 ratios are thought to accelerate the prion-like aggregation of Aβ, setting off the amyloid cascade.^6^ Previous work demonstrates that distinct fAD-causing mutations in PSEN1 and PSEN2 display characteristic Aβ processing stoichiometry, which has been hypothesized to drive variation in age at disease onset.^7^^,^^22^^,^^27^ In addition, patient-derived iPSC-neuron-secreted Aβ species have been shown to correlate with pTau/total ratio in a cohort of AD-focused iPSC lines.^1^

We found that both sAD and fAD iPSC-neurons display higher pTau/total tau ratios compared to control iPSC-neurons. However, the sAD group did not display significantly higher Aβ42/40 or Aβ43/40 ratios compared to controls, suggesting that additional non-Aβ-dependent mechanisms may alter pTau levels in these patients. Despite this, our results show a positive correlation between iPSC-neuron-secreted Aβ42/40 ratio and pTau/total tau ratio in the combined set of control, sAD, fAD, and DS cases. This effect appears to be driven primarily by the sAD and control groups. Notably, the fAD subgroup showed a non-significant negative correlation between Aβ42/40 ratio and pTau/total tau levels. This may be due to the increased production of Aβ43 at the expense of Aβ42 in some of these cell lines. Taken together, these data are consistent with the amyloid hypothesis of AD pathogenesis. Furthermore, they suggest that donor-dependent variation in Aβ processing and tau phosphorylation can be modeled in sAD iPSC-neurons, pointing the way toward studying patient-specific factors in AD pathogenesis. Future experimental manipulation of Aβ processing will be necessary to further investigate these findings. Further understanding the drivers of variation in Aβ processing and tau phosphorylation in iPSC-neurons may lead to new methods of screening for or modifying AD risk. Emerging anti-amyloid and anti-tau therapies entail considerable cost and may drive innovation in this area.^33–36^

Although the main focus of the study is on AD iPSC neurons, we also included cases with DS and FTLD-Tau as comparators. Increased Aβ secretion has been reported in iPSC-neurons and organoids derived from patients with DS.^37^^,^^38^ While our findings did not replicate this observation, there was a non-significant increase in Aβ40 and Aβ42 production in the DS lines as a group. In addition, our method of producing neurons differs from previously published results and may underlie the weaker phenotype observed in this work. Interestingly, of the two MAPT P301L mutant lines used in this study, only the line derived from a patient with primarily neuronal pathology showed increased pTau/total tau ratio (classical FTLD-Tau pathology vs CBD pathology). Future work will focus on understanding these phenotypes.

While the focus here is on neurons derived from a subset of 24 iPSC lines, our group has generated ∼500 primary dermal fibroblast lines from brain donors at the time of autopsy. We anticipate that the cell bank will have broad utility for researchers interested in neurodegenerative disease. Although neurons differentiated from iPSCs are of considerable use for research, a wide range of other disease-relevant cell types can also be generated from both iPSCs and fibroblasts. For example, iPSC-derived astrocytes^39–41^ and microglia^42^ are each being used as models to study pathogenesis in AD. In addition, neurons directly converted from fibroblasts are thought to maintain epigenetic age, unlike neurons differentiated from iPSCs.^43^^,^^44^ This feature may be an advantage for studying age-related phenotypes.

In summary, this work explores Aβ processing and tau phosphorylation in a panel of patient-derived iPSC-neurons and matched donor brains. We established correlations between the Aβ43 species produced by iPSC-neurons and those present in matched donor brains from fAD patients as well as alterations in Aβ processing in fAD iPSC-neurons. We also observed ApoE genotype as a potential modifier of Aβ processing in sAD iPSC-neurons. Finally, we demonstrated increased pTau levels in AD and fAD iPSC-neurons and identified a correlation between increased longer-length Aβ fragments and tau phosphorylation in these cells.

## Supplementary Material

nlae053_Supplementary_Data
