# Supplementary material for: β-Amyloid species production and tau phosphorylation in iPSC-neurons with reference to neuropathologically characterized matched donor brains
Source: J Neuropathol Exp Neurol. 2024 Jun 14;83(9):772–82. doi: 10.1093/jnen/nlae053 (PMC11333826; doi:10.1093/jnen/nlae053)
Supplement: nlae053_Supplementary_Data [file nlae053_supplementary_data.zip › nlae053_Supplementary_Data/Figure S2.pdf]

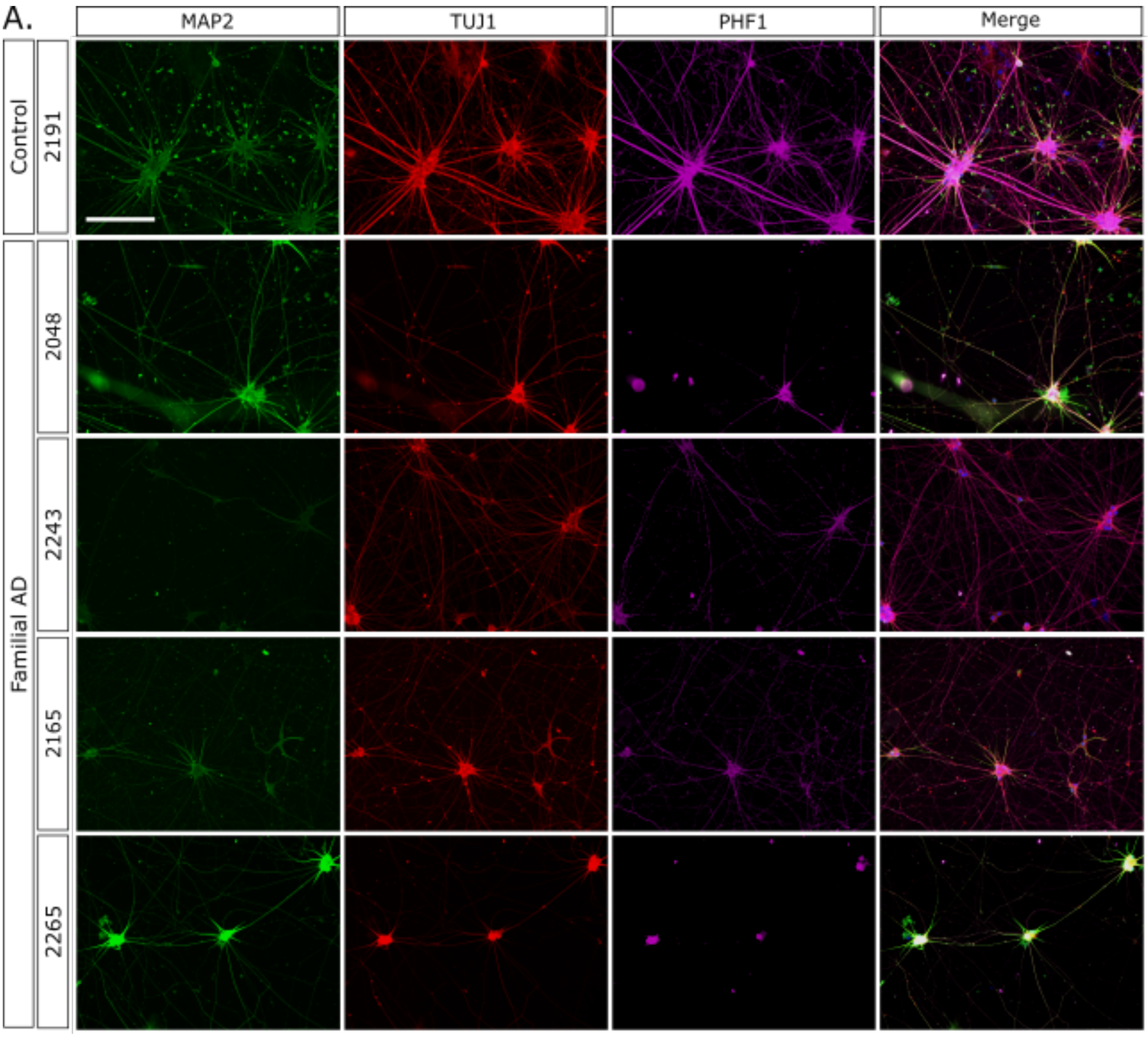

Figure S2: Neuronal differentiation of control and fAD iPSC lines (Lentiviral NGN2 integration). Immunohistochemistry for MAP2, TUJ1, and PHF1 phospho-tau in Day 28 neuronal cultures of each iPSC line, merged image counterstained with DAPI in blue. Scale bar = 100 microns.
