# Supplementary material for: β-Amyloid species production and tau phosphorylation in iPSC-neurons with reference to neuropathologically characterized matched donor brains
Source: J Neuropathol Exp Neurol. 2024 Jun 14;83(9):772–82. doi: 10.1093/jnen/nlae053 (PMC11333826; doi:10.1093/jnen/nlae053)
Supplement: nlae053_Supplementary_Data [file nlae053_supplementary_data.zip › nlae053_Supplementary_Data/Figure S3.pdf]

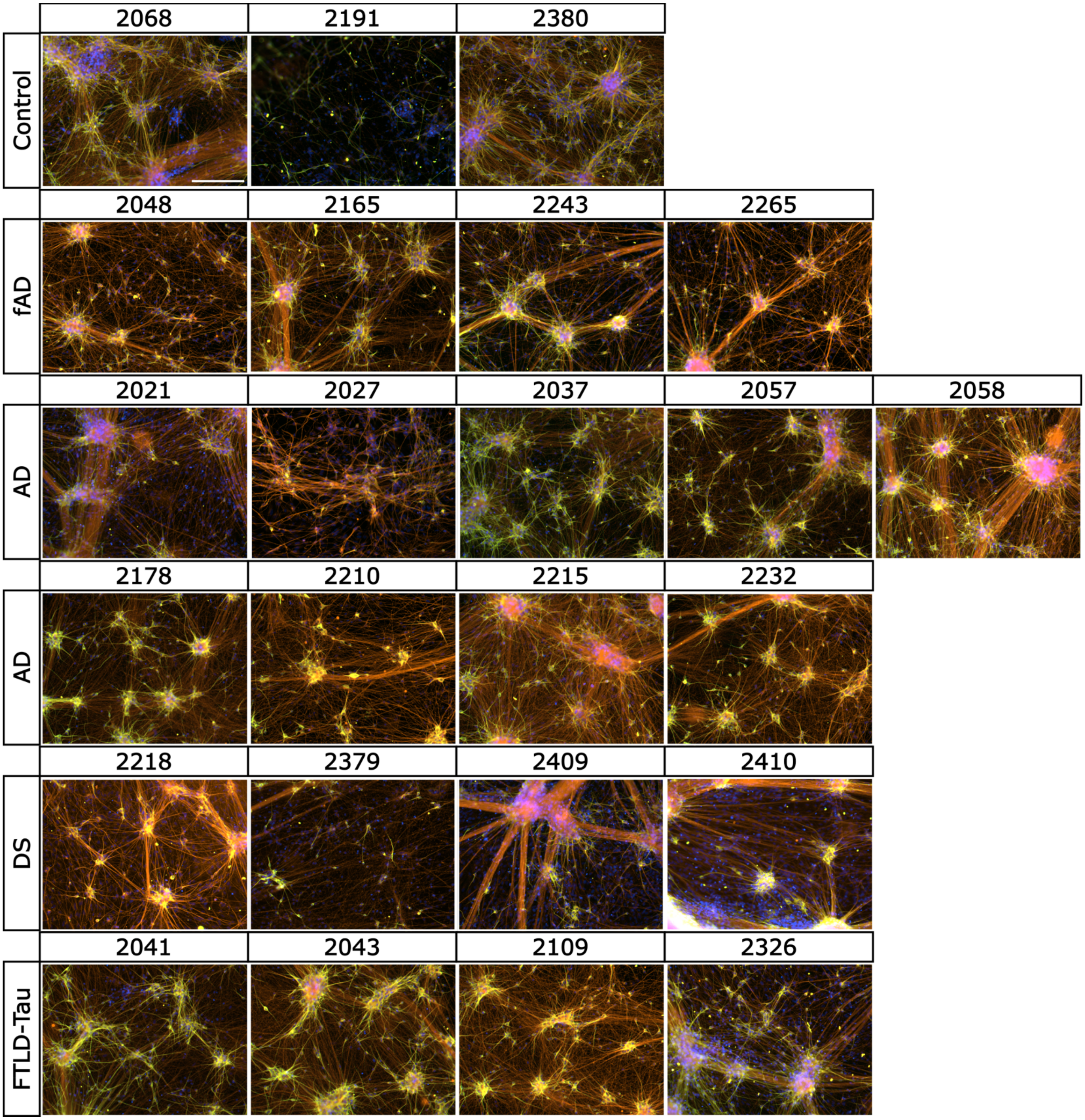

Figure S3: Neuronal differentiation of iPSC lines (PiB-NGN2 transposon integration). Merged images of immunohistochemistry for MAP2 (green) and TUJ1 (red) in Day 28 neuronal cultures of each iPSC line, counterstained with DAPI in blue. Scale bar = 200 microns
