# Supplementary material for: β-Amyloid species production and tau phosphorylation in iPSC-neurons with reference to neuropathologically characterized matched donor brains
Source: J Neuropathol Exp Neurol. 2024 Jun 14;83(9):772–82. doi: 10.1093/jnen/nlae053 (PMC11333826; doi:10.1093/jnen/nlae053)
Supplement: nlae053_Supplementary_Data [file nlae053_supplementary_data.zip › nlae053_Supplementary_Data/Figure S4.pdf]

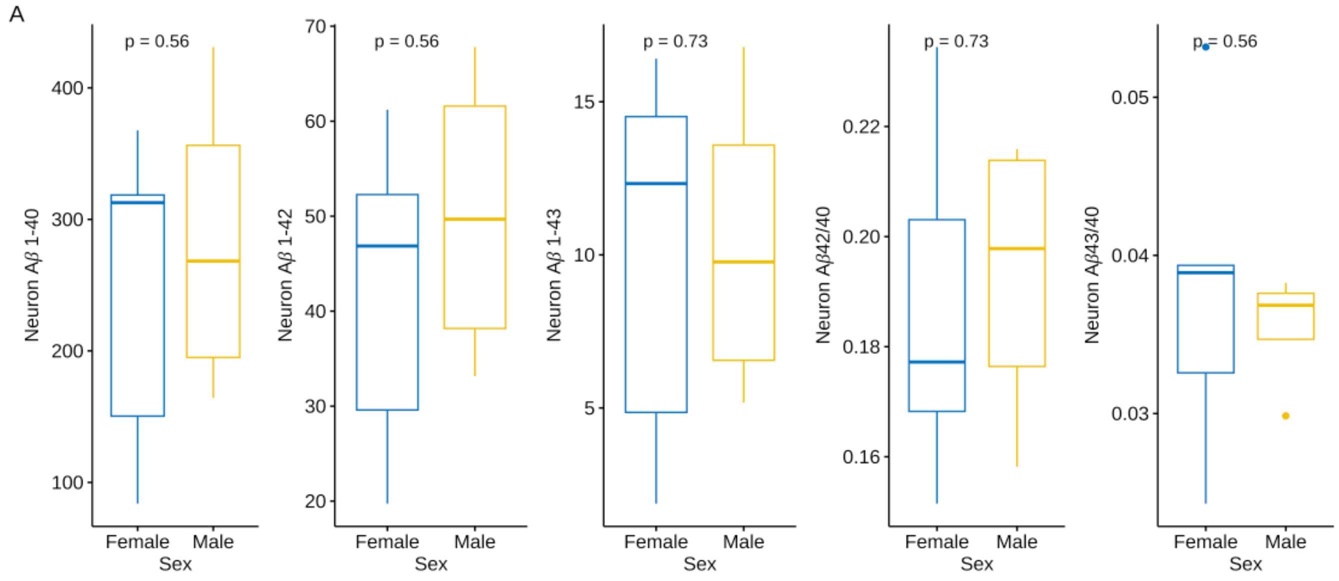

Figure S4: sAD donor sex does not influence neuronal Aβ species. A) Aβ species levels and ratios for female and male sporadic AD cases.
