# Supplementary material for: β-Amyloid species production and tau phosphorylation in iPSC-neurons with reference to neuropathologically characterized matched donor brains
Source: J Neuropathol Exp Neurol. 2024 Jun 14;83(9):772–82. doi: 10.1093/jnen/nlae053 (PMC11333826; doi:10.1093/jnen/nlae053)
Supplement: nlae053_Supplementary_Data [file nlae053_supplementary_data.zip › nlae053_Supplementary_Data/Figure S5.pdf]

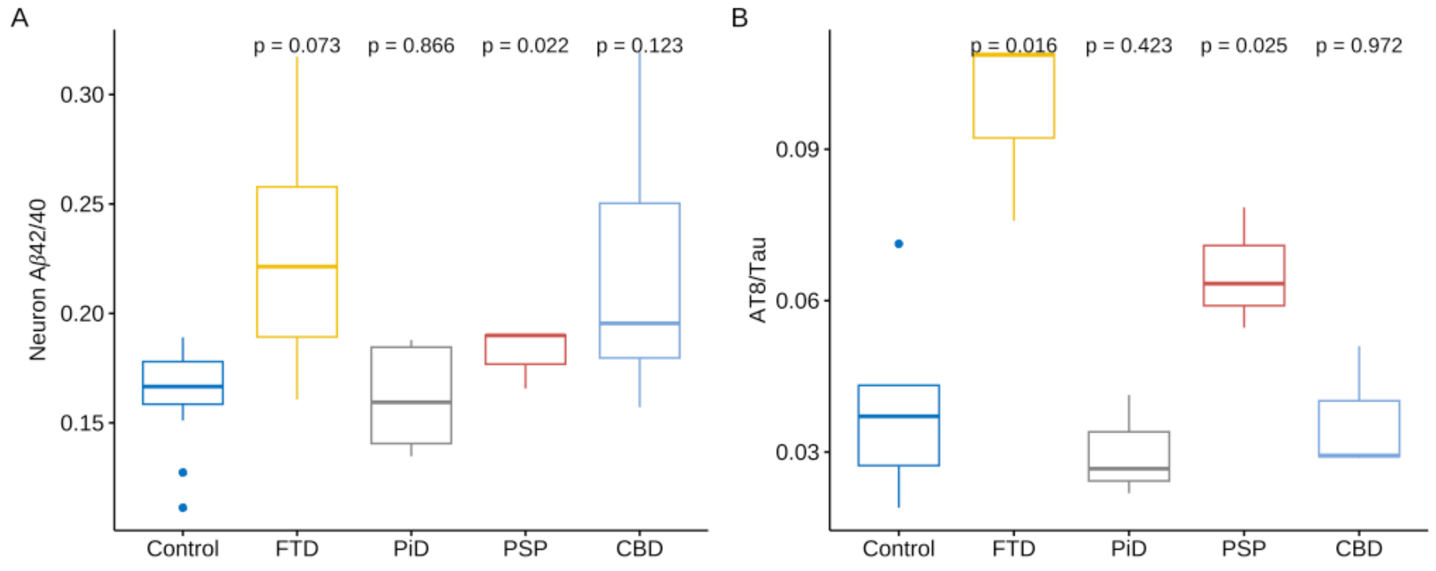

Figure S5: A subset of FTLD-Tau lines exhibit alteration in tau phosphorylation and Aβ production. Aβ42/40 ratio (A) and AT8/Total Tau ratios (B) for each FTLD-tau case compared to aggregate data for all 3 control lines. p values are listed above plot for comparison to control group. FTD=FTLD-tau with MAPT P301L mutation, PiD = Pick disease, PSP = progressive supranuclear palsy, CBD = corticobasal degeneration with MAPT P301L mutation.
